# Repressor element-1 silencing transcription factor regulates glutamate receptors and immediate early genes to affect synaptic plasticity

**DOI:** 10.18632/aging.203118

**Published:** 2021-06-09

**Authors:** Chenhaoyi Xu, Min Zhang, Lu Zu, Pei Zhang, Letao Sun, Xueyuan Liu, Min Fang

**Affiliations:** 1 Department of Neurology, Shanghai Tenth People's Hospital, Tongji University School of Medicine, Shanghai 200072, China; 2 Gordon F. Derner School of Psychology, Adelphi University, New York, NY 11530-0701, USA

**Keywords:** repressor element-1 silencing transcription factor, immediate early genes, synaptic plasticity, synaptic genes

## Abstract

Objective: This study aimed to investigate the regulatory effects of repressor element-1 silencing transcription factor (REST) on the glutamate receptors and immediate early genes (IEGs) in the SH-SY5Y cells.

Methods: The genes regulated by REST were screened by bioinformatics between AD patients and the control group. Then, SH-SY5Y cells were treated with 10 μM Aβ or REST siRNA/cDNA, and the expressions of synaptic genes and IEGs were detected. Moreover, the protein expression of synaptophysin and PSD-95 was detected by Western blotting in the primary mouse hippocampal neurons.

Results: Firstly, 464 differentially expressed genes regulated by REST were identified between Alzheimer’s disease (AD) patients and controls, and REST was closely related to the glutamatergic synapses and long-term potentiation. GRIA1, GRIN2A, GRIN1, and ARC showed significant variations with the changes of REST. Moreover, the loss of REST reduced the expression of synaptophysin and PSD-95, which was related to synaptic plasticity.

Conclusion: REST maintains synaptic plasticity by affecting both glutamate receptors and IEGs, and the imbalance between neural excitation and inhibition mediated by REST compromises neural function, contributing to cognitive impairment.

## INTRODUCTION

Neurons engage in the processes of capturing, encoding, and saving information, to learn and form memories [1]. It has been widely accepted that synapses can transmit information between neurons through electronic signals, chemical signals, and electronic-chemical signals [2]. Affected by the dynamic activity of neurons, the structure of synapses can change specifically within seconds to dynamically regulate their ability to release neurotransmitters [1]. This close relationship between structure and function is regarded as synaptic plasticity. Once neurons are activated, numerous proteins are synthesized subsequently through a series of signaling pathways for the maintenance of homeostatic synaptic plasticity, and the synthesis and translation of the related proteins are important to maintain the synaptic plasticity [3]. For example, glutamate AMPA type subunit1 (GRIA1), glutamate NMDA type subunit 1 (GRIN1), and glutamate NMDA type subunit 2A (GRIN2A) can encode glutamate AMPA receptor and glutamate NMDA receptor, respectively, which then bind to glutamate released from the pre-synapses, to respond to long-term potentiation (LTP) - inducing or long-term depression (LTD) - inducing stimuli [4]. The imbalance among the associated proteins involved in the transcription, translation, and degradation may cause the abnormality of neural circuits, finally resulting in neurodegenerative diseases, such as Alzheimer’s disease (AD), Parkinson’s disease, and others [5].

Synaptic plasticity participates in the informational storage in the brain, and it can be regulated by a number of factors, including repressor element-1 silencing transcription factor (REST). REST can act on synaptic genes and immediate early genes (IEGs) to repress neural activity and avoid hyperexcitation in the aging brain [6, 7]. REST, a zinc-finger transcription factor, mainly binds to repressor element-1 (RE-1) motifs of the target genes to repress gene expression, and some studies also reveal that REST may also act as a transcriptional promoting factor [8]. In the post-natal and aging brains, the REST expression remains at a high level to ensure neuronal specificity and cell type-specific gene expression. The regulatory effects of REST are complicated due to its various binding sites on the target genes and its environment-dependence which is influenced by the locations and types of cells [9]. Studies have shown that the reduced REST expression in aging adults is involved in the pathogenesis of AD. However, how REST regulates the epigenetic modification of targeted mRNAs remains largely unknown. In our previous studies, our results showed high Aβ load reduced REST expression [10]. This study aimed to explore the regulatory effects of REST on the synaptic genes and IEGs, which may provide evidence on the role of REST in synaptic plasticity and on the potential mechanism underlying the initiation of AD.

IEGs, such as activity-regulated cytoskeleton-associated protein (ARC), neuronal Per/Arnt/Sim domain protein 4 (NPAS4), early growth response protein 1 (EGR1), and brain-derived neurotrophic factor (BDNF), are subsets of genes induced by stimuli in seconds [11]. In neurons, IEGs can be induced immediately and play critical roles in the establishment and maintenance of permanent changes of neurons, forming learning and memory [12]. The abnormal expression of IEGs may lead to the occurrence of cognitive disorders, schizophrenia, and other diseases [13]. Particularly, the induction of ARC is associated with the consolidation process of labile memories through its ability of prior history of neuronal activation to facilitate LTP or LTD, which has been termed as metaplasticity to influence synaptic plasticity [14]. It has been revealed that the ARC expression reduces in the dentate gyrus and CA1 region of AD mice, and the neurons with amyloid plaque-associated dystrophic neurites fail to express ARC mRNA [15].

We hypothesize that REST may regulate synaptic genes and IEGs to affect the synaptic plasticity, and Aβ can decrease REST expression, ultimately affecting the synaptic plasticity to cause the abnormal excitability of the neural network.

## MATERIALS AND METHODS

### Cell culture and transfection

Human neuroblastoma SH-SY5Y cells were cultured in Dulbecco’s Modified Eagle’s Medium (DMEM) containing 10% Fetal Bovine Serum (FBS), 1% penicillin-streptomycin (PS) at 37°C with a mixed gas of 21% O_2_, 5% CO_2_, and N_2_.

Primary mouse hippocampal neurons were collected from neonatal C57BL/6 mice as described previously [16]. Then, 6 × 10^5^ cells were plated in 6-well plates coated by 0.05 mg/ml poly-L-lysine (PLL, BBI Life Science Corporation, China) and incubated with B-27 Serum-Free Supplement (Gibco, USA), GlutaMAX Supplement (Gibco, USA) and Neurobasal Medium (Gibco, USA) at 37°C with a mixed gas of 21% O_2_, 5% CO_2_ and N_2_. For the Aβ treatment, cells were incubated with 10 μM human Aβ_1-42_ (MCE, China) for 48 h.

For transfection, SH-SY5Y cells or primary mouse hippocampal neurons were plated in 6-well plates at the density of 3 × 10^5^ cells/well. REST siRNA (Zorin Bio, Shanghai, China) was transfected into cells with Lipofectamine 3000 kit according to the manufacturer’s instructions (Thermo Fisher, USA). The target sequence of REST siRNA was GCGTACTCATTCAGGTGAGAA.

### RNA isolation and real-time quantitative polymerase chain reaction (RT-qPCR)

Total RNA was extracted by Trizol reagent and transcribed reversely into cDNA using the PrimeScript RT reagent kit (Takara Bio, Japan). Real-time PCR was performed using the TB Green Premix Ex Taq II kit (Takara Bio, Japan). The primers were designed in Primer Premier 5.0 software (Premier, Canada) and synthesized by Sangon Bio (Shanghai, China). The sequences of primers are listed in the Supplementary Materials. The reaction conditions were as follows: 95°C for 30 s, and 50 cycles of 95°C for 3 s and 60°C for 30 s. The mRNA expression of target genes was normalized to that of β-actin as the internal reference.

### Western blotting

SH-SY5Y cells were treated by RIPA Lysis Buffer (Epizyme, China) with 1 mM PMSF (Beyotime, China) on ice for 5 s. Then, the lysate was centrifuged at 14,000 g for 5 min. The supernatant was collected and the concentration of total protein was quantified by bicinchoninic acid assay (Beyotime, China). 15 μg of proteins were loaded and separated on 10% SDS-PAGE. Then, proteins were transferred onto PVDF membranes which were blocked with 5% non-fat milk in TBST buffer at room temperature for 1 h. The membranes were incubated with appropriate primary antibodies at 4°C overnight and then with corresponding secondary antibodies. The optical density of protein bands was quantified using Image J. Antibodies used in experiments were as follows: REST (Abcam, #ab75785), ARC (Proteintech, #16290-1-AP), synaptophysin (Abcam, #ab52636), PSD-95 (Abcam, #ab18258), GAPDH (BBI, #D110016), TUBULIN (Affinity, #AF7011), goat anti-rabbit IgG H&L (Abcam, #ab6721), and goat anti-Mouse IgG H&L (Abcam, #ab205719).

### Chromatin immunoprecipitation and polymerase chain reaction (ChIP-PCR)

ChIP was performed using the ChIP Assay kit (Beyotime, China) according to the manufacturer’s instructions. In short, cells were pretreated by ultrasound to maintain DNA–protein complexes and then immunoprecipitated with ChIP grade antibodies against REST (Sigma-Aldrich, #17–641) or IgG. DNA obtained from the immunoprecipitation was further analyzed by PCR.

### Statistical analysis

All the data are expressed as mean ± standard error (SEM). All experiments were independently repeated at least three times. Data were analyzed in SPSS version 22.0 software (IBM, USA). All the data were in accordance with Gaussian distribution as shown by Shapiro-Wilk’s test. Subsequently, Levene’s test was used to test the equality of variances. The equal-variances data between the two groups were compared with the two-tailed student’s *t*-test and the unequal-variances data with the corrected two-tailed student’s *t*-test. Comparisons of data among 3 or more groups were done with the analysis of variance (ANOVA). A value of *p* < 0.05 was considered statistically significant.

### Ethical statement

This study was approved by the local ethics committee of Tenth People’s Hospital, Tongji University School of Medicine, Shanghai (No. SHDSYY-2016-2722). All the procedures were done according to the ARRIVE guidelines. The authors are accountable for all aspects of the work in ensuring that questions related to the accuracy or integrity of any part of the work are appropriately investigated and resolved.

## RESULTS

### Identification of synapsis related genes regulated by REST

**Figure 1:**
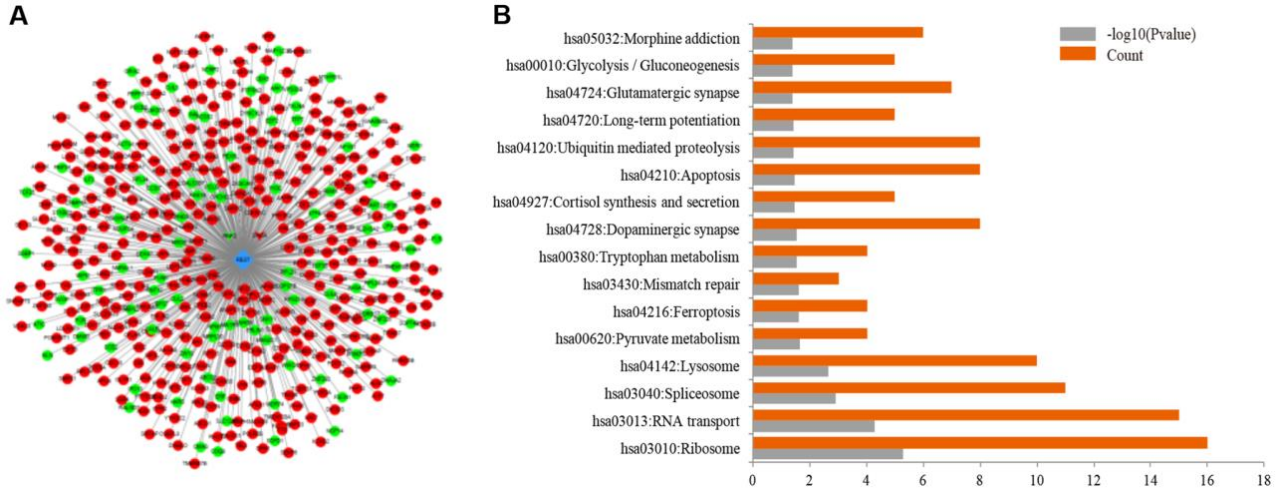
**Prediction of related genes associated with REST.** (**A**) ENCODE database included 464 differentially expressed transcripts related to REST, including 2 upstream genes and 462 downstream genes. The rhomboid nodes represented REST, the v-shaped nodes were the upstream genes of REST, and the circular nodes were the downstream genes of REST. The red nodes represented up-regulated genes by REST and green nodes represented down-regulated genes by REST. (**B**) For the differentially expressed genes regulated by REST, synaptic functions, such as glutamatergic synapses, LTP, dopaminergic synapses, were significantly enriched as shown in the GO analysis.

Encyclopedia of DNA Elements (ENCODE, https://www.encodeproject.org) record the results from different ChIP-seq, thus the upstream and downstream genes of REST were predicted in ENCODE, and a total of 26 upstream genes and 6587 downstream genes were identified. Then, the differential transcripts regulated by REST were screened in these 6613 genes. Results showed there were 2 upstream genes and 462 downstream genes of REST were differentially expressed. As shown in Figure 1A, the rhomboid nodes represented REST, the v-shaped nodes were the upstream genes of REST, the circular nodes were the downstream genes of REST, the red nodes referred to the up-regulated genes by REST and green nodes were the down-regulated genes by REST (Figure 1A and Supplementary Tables 1 and 2). Further gene ontology (GO) analysis was performed by cluster Profiler to identify the functions of these 464 differential genes. Interestingly, synaptic functions associated with GO terms, such as glutamatergic synapses, LTP, and dopaminergic synapse, showed significantly enriched (Figure 1B), suggesting that REST is involved in the synaptic functions.

### Knockdown of REST reduced synaptophysin and PSD-95 expression in primary mouse hippocampal neurons

**Figure 2:**
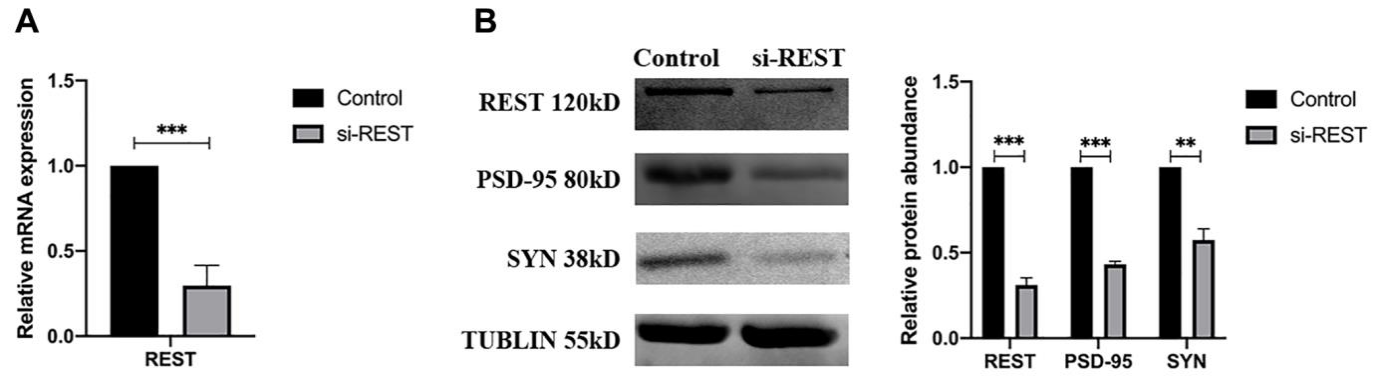
**REST knockdown reduced the synaptophysin (SYN) and PSD-95 expression in the primary mouse hippocampal neurons.** (**A**) REST was successfully knocked down in the primary mouse hippocampal neurons. *n* = 3. (**B**) SYN and PSD-95 was deregulated after knockdown of REST. *n* = 3. Independent experiments were performed three times. Data are expressed as mean ± SEM. ^*^p < 0.05.

The primary mouse hippocampal neurons were separated from neonatal C57BL/6 mice and then transferred with REST siRNA. The successful knockdown of REST was verified by qPCR (Figure 2A). To clarify the phenotypes of synaptic plasticity, western blotting was performed. Results showed that the expression of synaptophysin (SYN) and PSD-95 (two synaptic biomarkers) was reduced significantly after the knockdown of REST (Figure 2B).

### Effects of REST on mRNA of synaptic genes

**Figure 3:**
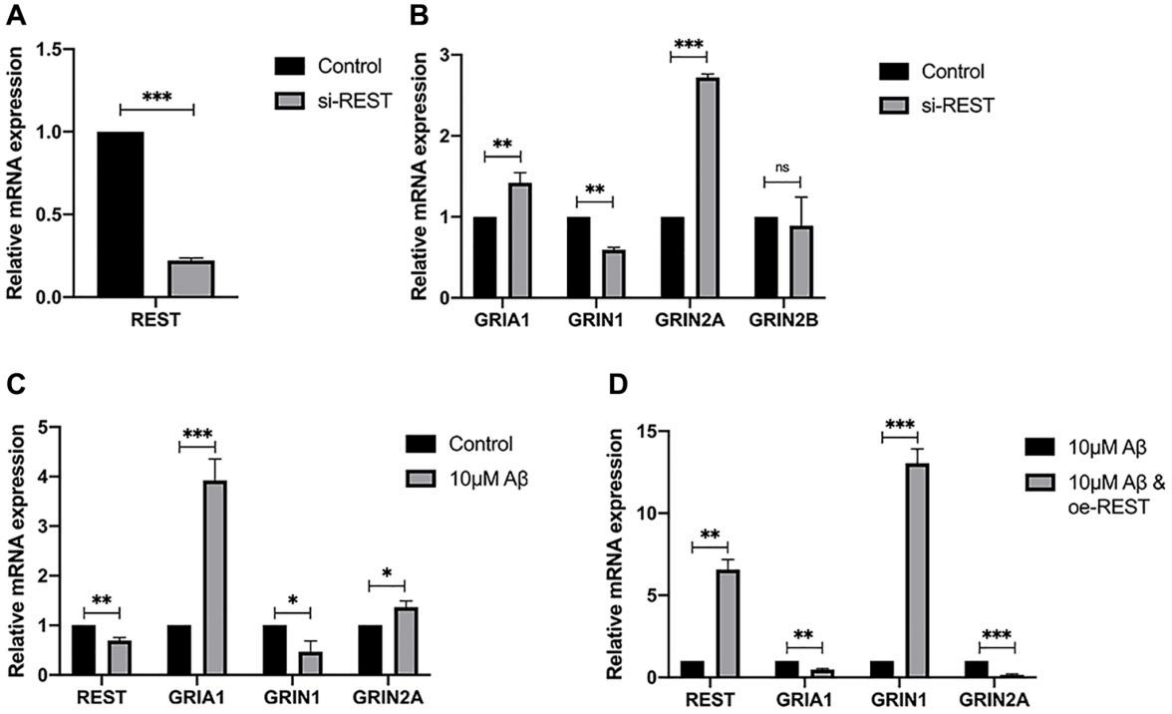
**Effects of REST on the expression of synaptic genes.** (**A**) REST was successfully knocked down in SH-SY5Y cells. *n* = 3. (**B**) REST knockdown increased GRIA1 and GRIN2A expression, while declined GRIN1 expression in SH-SY5Y cells. *n* = 3. (**C**) 10 μM Aβ increased the mRNA expression of GRIA1 and GRIN2A, and decreased GRIN1 mRNA expression. *n* = 3. (**D**) REST over-expression significantly increased the mRNA expression of GRIN1, which was decreased by 10 μM Aβ in SH-SY5Y cells. *n* = 3. Independent experiments were performed three times. Data are expressed as mean ± SEM. ^*^p < 0.05, ^**^p < 0.01 and ^***^p < 0.001 vs the corresponding control group.

The REST expression was knocked down in SH-SY5Y cells, aiming to verify the results of ENCODE and explore the genes regulated by REST (Figure 3A). qPCR results showed that REST knockdown reduced the mRNA expression of GRIN1, but increased the mRNA expression of GRIA1 and GRIN2A (Figure 3B). Then, cells were incubated with 10 μM Aβ for 48 h. After REST knockdown, the mRNA expression of GRIN1 decreased but that of GRIA1 and GRIN2A increased (Figure 3C). Moreover, the GRIN1 mRNA expression was rescued after over-expressing REST (Figure 3D).

### Effects of REST on IEGs

**Figure 4:**
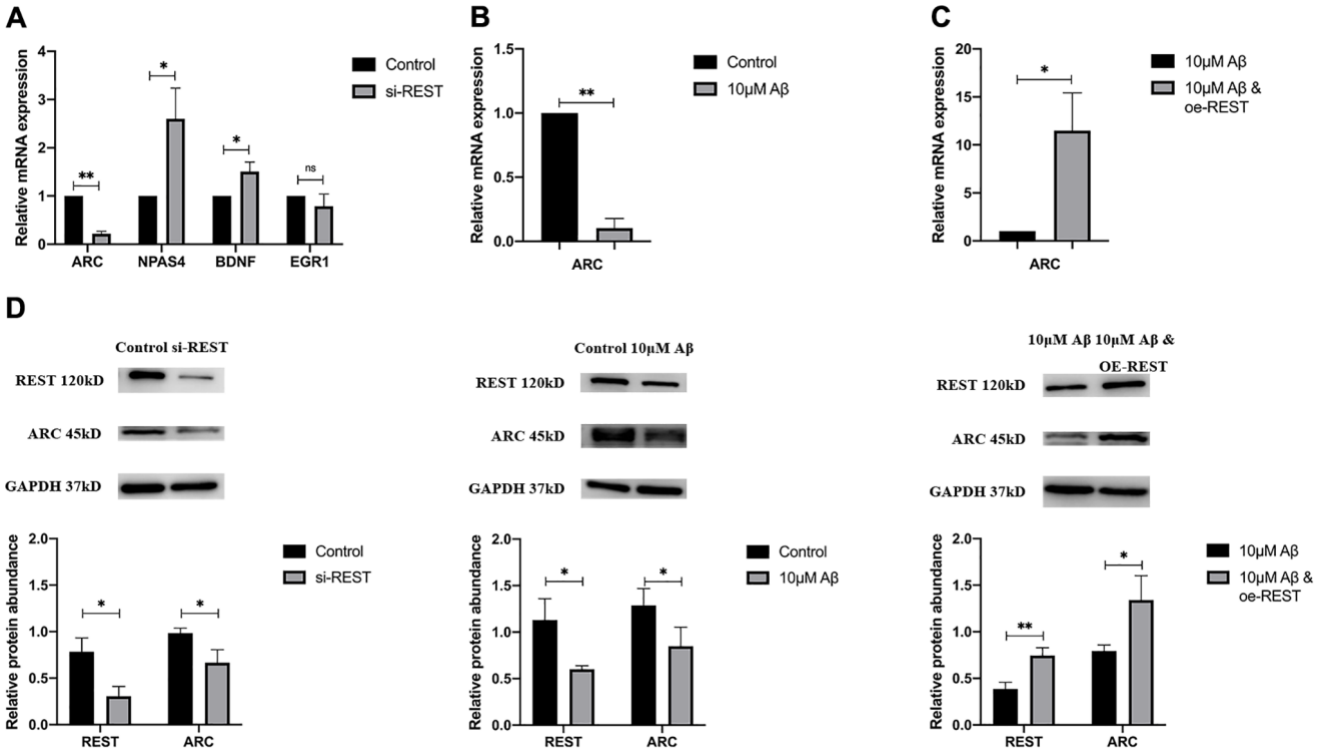
**Effects of REST on IEGs.** (**A**) REST knockdown declined ARC expression, while increased NPAS4 and BDNF expression in SH-SY5Y cells. *n* = 3. (**B**) 10 μM Aβ decreased ARC mRNA expression. *n* = 3. (**C**) Over-expression of REST significantly increased ARC mRNA expression, which was decreased by 10 μM Aβ. *n* = 3. (**D**) The ARC protein expression showed a similar trend to its mRNA expression. *n* = 3. Independent experiments were performed three times. Data are expressed as mean ± SEM. ^*^p < 0.05, ^**^p < 0.01 and ^***^p < 0.001 vs the corresponding control group.

IEGs are also related to synaptic functions. In our study, the mRNA expressions of IEGs were detected in SH-SY5Y cells after the REST knockdown. Results indicated that REST knockdown could improve the mRNA expression of NPAS4 and BDNF (Figure 4A), which was consistent with previous findings [9]. In addition, the ARC mRNA expression decreased in the case of REST knockdown (Figure 4A). Aβ treatment also declined the ARC mRNA expression in SH-SY5Y cells, which could be rescued by over-expressing REST (Figure 4B and 4C). The ARC protein expression showed a similar trend to its mRNA expression (Figure 4D).

### Inhibition of combination between REST and ARC by Aβ

**Figure 5:**
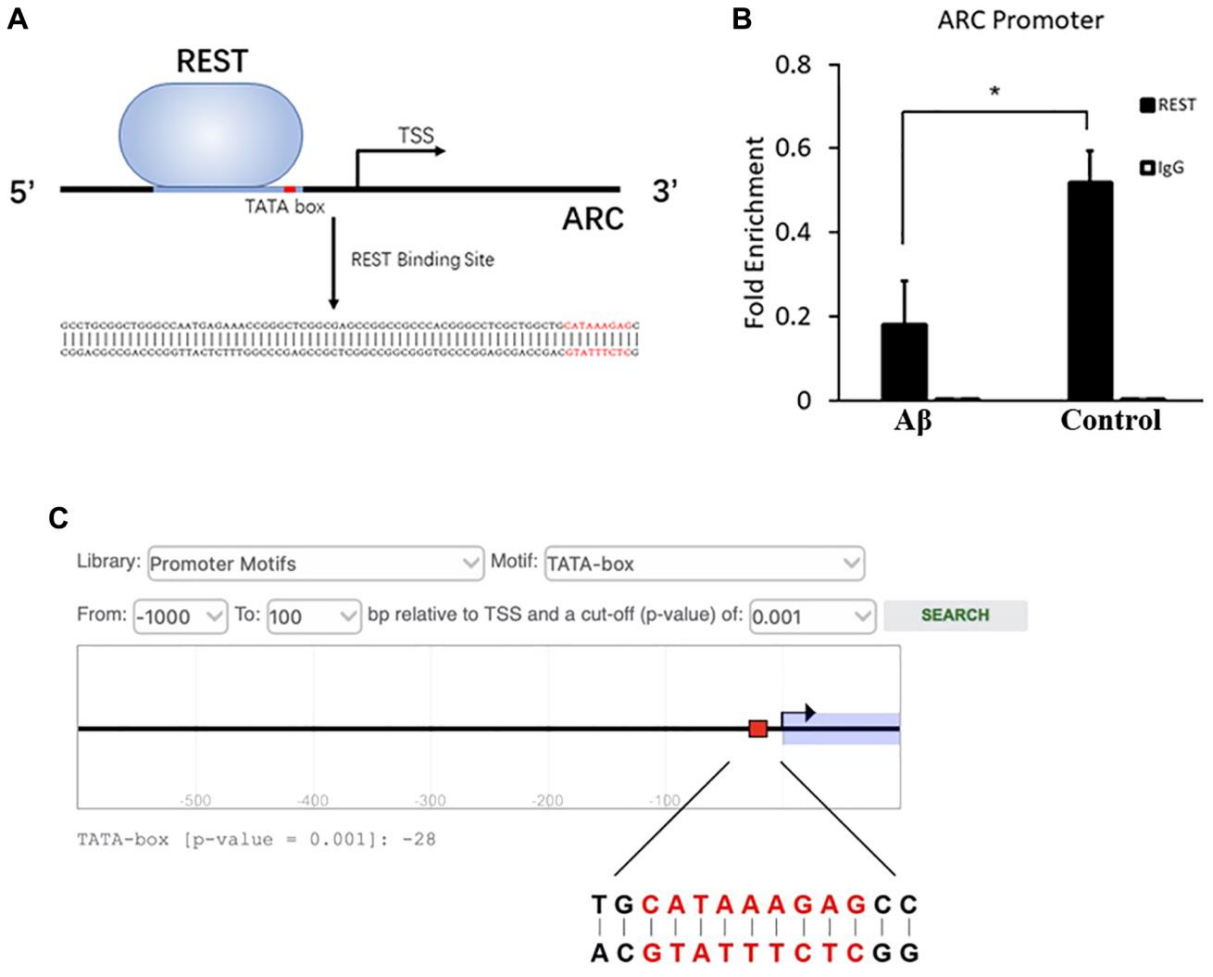
**Inhibition of combination between REST and ARC by Aβ.** (**A**) The REST-binding site at -75 to -19 bp of ARC gene. (**B**) Aβ reduced the interaction between REST and ARC. *n* = 3. (**C**) The TATA-box of ARC at -28 bp as shown by the prediction of EPD. Independent experiments were performed three times. Data are expressed as mean ± SEM. ^*^p < 0.05 vs the corresponding control group.

ChIP-PCR was performed in SH-SY5Y cells, aiming to investigate the interaction of REST and ARC. Results showed a REST-binding site at -75 to -19 bp of ARC gene (Figure 5A). After Aβ treatment, the combination between REST and ARC reduced as compared to that in the control group (Figure 5B). In addition, the ARC sequence was analyzed in the Eukaryotic Promoter Database (EPD, https://epd.epfl.ch/index.php), and results showed a TATA box at -28 bp of ARC gene (Figure 5C). We speculated that REST might influence TATA-box on the ARC gene, thus initiating its transcription to directly activate the ARC expression.

## DISCUSSION

This study indicated that REST could regulate synaptic genes and IEGs to affect synaptic plasticity. On the one hand, REST up-regulated the mRNA expression of GRIN1 and down-regulated the mRNA expression of GRIA1 and GRIN2A; on the other hand, REST transcriptionally activated ARC, which may lead to the loss of GRIA1, due to the GRIA1 internalization through ARC. REST could buffer the changes in the neural excitation to maintain synaptic plasticity, and the loss of REST could lead to the imbalance between neural excitation and inhibition.

### Different effects of REST on glutamate receptors

The induction of REST is common in the normal aged human cortical and hippocampal neurons, but REST expression is down-regulated in the brain of AD patients [7]. This phenomenon may attribute to the loss of REST from the nucleus and its appearance in autophagosomes. As a transcriptional factor, REST may differentially regulate numerous genes depending on the external environments, cell locations, and cell types. In the present study, results showed the differentially expressed genes associated with REST were related to glutamatergic potentiation as shown by GO analysis (Figure 1A and Figure 1B). Glutamate receptors are essential for the formation of synaptic plasticity, and the expression of synaptophysin and PSD-95 is closely associated with glutamate receptors. To observe the phenotype, primary neurons were used to reflect the changes of synapse. The expression of two synaptic biomarkers, synaptophysin and PSD-95, declined after REST knockdown in the primary mouse hippocampal neurons (Figure 2). To further investigate the mechanism, we chose SH-SY5Y cells, which were used as typical cells for the research of mechanism, for the following studies. Results showed the expression of GRIN2A and GRIA1 declined by REST, while GRIN1 expression was positively related to REST expression (Figure 3A, Figure 3B, and Figure 3C). Glutamate receptors reflect the function of synapse. GRIN1 and GRIN2A genes can encode glutamate NMDA receptors, GRIA1 can encode glutamate AMPA receptors, and glutamate (a neurotransmitter in the central nervous system) may bind to these receptors to affect synaptic plasticity. GRIN2A and GRIN1 had been reported as the repressed target genes of REST [6, 17]. In addition, REST has been described to repress the expression of numerous genes. Besides, REST may also function as an activator to induce the proliferation of neurons [18]. Our findings were inconsistent with previous findings that GRIN1 expression was repressed by REST in the ischemia-induced neuronal death model [17]. This discrepancy might be explained as follows. First, the function of REST is closely dependent on the cell types and context-specific. In addition, there is more than one RE-1 motif on the target genes, and the location of binding sites, including the regulatory regions or exons of target genes, may directly determine the different regulatory effects by REST. Moreover, the N/C-terminal domains of REST may interact with various transcriptional factors, directly or indirectly alternating the expression of target genes [9]. Therein, although GRIN1 expression declines after REST up-regulation in the ischemic stroke, the different circumstances, especially the different cell types and cultural environments, might lead to this discrepancy. Also, our results showed the loss of REST increased the GRIA1 expression. However, GRIA1 hasn’t been identified as a target gene of REST. Thus, we speculate REST might down-regulate the expression of GRIA1 through other mechanisms, and REST might influence the synaptic function partly via its effects on the GRIA1, GRIN1, and GRIN2A. Among them, GRIN1 is important for the basic function of NMDA receptors; GRIN2A is a regulatory subunit of NMDA receptors and GRIN2A expression can be induced to form synaptic plasticity [19]. Under high Aβ load, the loss of REST leads to the imbalance of glutamate receptors, finally influencing synaptic plasticity.

### REST may affect on IEGs, especially via the direct activation of ARC

The abnormal expression of IEGs is also related to the imbalance of synaptic plasticity. IEGs can engage in the signal cascade to alternate the expression of important genes for the consolidation of long-term memory to stabilize LTP [20]. The expression of NPAS4 and BDNF was repressed by REST, which is consistent with previous findings [21, 22], and the expression of EGR1 showed no change. Additionally, ARC expression is positively related to the REST expression (Figure 4A, to Figure 4D). To explore whether REST regulates the expression of ARC, ChIP-PCR was performed in SH-SY5Y cells. Results showed a REST-binding site at -75 to -19 bp of ARC gene, which included the TATA-box at -28 bp (Figure 5A). Thus, REST may affect TATA-box to initiate the transcription. Moreover, after Aβ treatment, the interaction between REST and ARC was reduced (Figure 5B). However, more experiments, such as electrophoretic mobility shift assay (EMSA) or/and dual-luciferase reporter assay, are needed to clarify the specific mechanism. Based on the above findings, we speculate that a high Aβ load may directly decrease ARC expression and indirectly reduce the transcriptional effects of REST by inhibiting the interaction between REST and ARC. The expression of ARC, also known as ARG3.1, experiences a highly dynamic process during memory formation [23]. ARC mainly interacts with cellular skeletal proteins to promote the internalization of AMPA receptors, which induces LTD and further regulates homeostatic synaptic scaling and synaptic plasticity [24]. Disordered ARC expression may cause an imbalance of cognitive flexibility, and thus neurons can not capture the new information [25]. Our results showed ARC expression was down-regulated after REST knockdown, which means the reduced ARC expression may weaken the GRIA1 internalization, resulting in the elevation of GRIA1 expression. Therein, we speculate that REST may activate ARC to indirectly down-regulate the GRIA1 expression.

In summary, REST may regulate the function and structure of synapses to affect synaptic plasticity. On one hand, REST may decrease GRIN2A and GRIA1 expression but activate GRIN1 expression to regulate the excitatory-inhibitory balance of neural circuits. On the other hand, REST may interact with ARC to indirectly alternate the long-term depression and influence synaptic scaling. The imbalance between neural excitation and inhibition mediated by REST may compromise the neural function, contributing to the aging process.

## Supplementary Materials

Supplementary Tables
