# Supplementary material for: Repressor element-1 silencing transcription factor regulates glutamate receptors and immediate early genes to affect synaptic plasticity
Source: Aging (Albany NY). 2021 Jun 9;13(11):15569–79. doi: 10.18632/aging.203118 (PMC8221361; doi:10.18632/aging.203118)
Supplement: Supplementary Tables [file aging-13-203118-s001.pdf]

SUPPLEMENTARY TABLES

Supplementary Table 1. Primers.

Gene	Primer	Sequence
β-ACTIN	Forward Primer	5′-CTTCGCGGGCGACGAT-3′
	Reverse Primer	5′-CCACATAGGAATCCTTCTGACC-3′
REST	Forward Primer	5′-AAACTTGAATACGCCAGAGGG-3′
	Reverse Primer	5′-GCCATTGTAGCAGGAGGTGAT-3′
GRIA1	Forward Primer	5′-GGCTACTCCTACCGTCTGGA-3′
	Reverse Primer	5′-AATTTGTCCAAAAGCCCCTGC-3′
GRIN1	Forward Primer	5′-CAACGACCACTTCACTCCCA-3′
	Reverse Primer	5′-GACACGCATCATCTCAAACC-3′
GRIN2A	Forward Primer	5′-CTTGGAAGAGGCAGATCGAC-3′
	Reverse Primer	5′-CTTCTCGTTGTGGCAGATCC-3′
GRIN2B	Forward Primer	5′-AGCTGCTCTCCATACCCTGA-3′
	Reverse Primer	5′-TCGTCGACTCCCTTGGTTTG-3′
ARC	Forward Primer	5′-TCATCCAGATGTGAGCACCG-3′
	Reverse Primer	5′-GATGGCCTGAGAGTGTGGAG-3′
NPAS4	Forward Primer	5′-GACCAGATCAACGCCGAGAT-3′
	Reverse Primer	5′-AGTGCCACCAGCGAAGAAGA-3′
BDNF	Forward Primer	5′-GTAAAGCCAACCCTGTGTCG-3′
	Reverse Primer	5′-TCCGCTCCAAAATCTGACTC-3′
EGR1	Forward Primer	5′-GGATCCTTTCCTCACTCGCC-3′
	Reverse Primer	5′-CGTTGCTCAGCAGCATCATC-3′
METTL3	Forward Primer	5′-TTGTCTCCAACCTTCCGTAGT-3′
	Reverse Primer	5′-CCAGATCAGAGAGGTGGTGTAG-3′
METTL14	Forward Primer	5′-AGTGCCGACAGCATTGGTG-3′
	Reverse Primer	5′-GGAGCAGAGGTATCATAGGAAGC-3′
FTO	Forward Primer	5′-ACTTGGCTCCCTTATCTGACC-3′
	Reverse Primer	5′-TGTGCAGTGTGAGAAAGGCTT-3′
YTHDF1	Forward Primer	5′-GACGACATCCACCGCTCCATT-3′
	Reverse Primer	5′-CCCACTCCCATTGACGCTGAAG-3′

Supplementary Table 2. The function of target genes.

ID	Description	*P* value	Gene ID	Count
hsa03010	Ribosome	5.10E-06	/RPL41/RPS7/RPL3/RPS15/FAU/MRPL30/RPS11/RPL30/RPL7A/RPL17/RPL34/RPL21/RPL28/RPS27A/RPL23/RPL27	16
hsa03013	RNA transport	5.39E-05	/NUPL2/MAGOHB/TRNT1/STRAP/EIF4G3/NUP85/PHAX/EEF1A1/EIF3B/CLNS1A/NCBP2/EIF3G/SEC13/EIF4A2/TPR	15
hsa03040	Spliceosome	1.27E-03	/MAGOHB/SNRNP70/TRA2B/RBMX/LSM6/SNRPC/USP39/NCBP2/HNRNPA1/PRPF8/TCERG1	11
hsa04142	Lysosome	2.24E-03	/AP1G1/GUSB/NPC1/GGA1/ATP6V0A1/GBA/LIPA/AP3B2/GGA3/CD63	10
hsa00620	Pyruvate metabolism	2.27E-02	/ALDH7A1/ACSS2/ALDH3A2/PDHA1	4
hsa04216	Ferroptosis	2.46E-02	/VDAC3/GCLC/MAP1LC3B/CP	4
hsa03430	Mismatch repair	2.52E-02	/PMS2/RFC5/SSBP1	3
hsa00380	Tryptophan metabolism	2.89E-02	/ALDH7A1/ALDH3A2/DHTKD1/AFMID	4
hsa04728	Dopaminergic synapse	3.00E-02	/ITPR1/COMT/GNB4/GRIN2A/GRIA2/ATF4/ITPR2/GNB2	8
hsa04927	Cortisol synthesis and secretion	3.46E-02	/ITPR1/PBX1/ATF4/ITPR2/PDE8A	5
hsa04210	Apoptosis	3.63E-02	/ACTG1/ITPR1/NFKBIA/SPTAN1/DIABLO/ATF4/ITPR2/CFLAR	8
hsa04120	Ubiquitin mediated proteolysis	3.77E-02	/CUL3/CDC27/ANAPC11/TRIM37/UBE2L3/CUL2/CDC16/UBE3B	8
hsa04720	Long-term potentiation	3.87E-02	/ITPR1/GRIN2A/GRIA2/ATF4/ITPR2	5
hsa04724	Glutamatergic synapse	4.00E-02	/ITPR1/GNB4/DLGAP1/GRIN2A/GRIA2/ITPR2/GNB2	7
hsa00010	Glycolysis / Gluconeogenesis	4.08E-02	/ALDH7A1/ACSS2/ALDH3A2/ALDOA/PDHA1	5
hsa05032	Morphine addiction	4.14E-02	/PDE2A/PDE1B/GNB4/GABRB3/GNB2/PDE8A	6
